# Multi-Focal, Multi-Centric Angiosarcoma of Bone

**DOI:** 10.1080/13577149778290

**Published:** 1997-12

**Authors:** Eleni Kakouri, Jeremy S. Whelan, Stewart Coltart, Mark E. F. Smith, Robert L. Souhami

**Affiliations:** 1 The London Soft Tissue & Bone Tumour Service The Meyerstein Institute of Oncology The Middlesex Hospital Mortimer Street London W1N 8AA UK; 2 Kent and Canterbury Hospital Canterbury Kent UK; 3 Department of Histopathology University College Hospital London UK

## Abstract

A multi-focal multi-centric, malignant tumour of vascular origin arising in bone in a 55-year-old man is described. The presenting symptoms were pain and weight loss. Radiologically, multiple lytic lesions were demonstrated in the long bones of both legs and throughout the pelvis. Histological examination demonstrated an angiosarcoma which was predominantly low grade in nature but with focal areas of intermediate grade. Turnout cells expressed the endothelial markers CD31, CD34 and von Willebrand's factor. There was rapid radiological progression of disease with no response to radiotherapy. Pain abated within a few days of institution of doxorubicin, 75 mg m^-2^, but the patient died of massive pulmonary thromboembolism 14 days later, 11 months after the first symptoms.


*Sarcoma* (1997) 1, 183–187

CASE REPORT

### Multi-focal, multi-centric angiosarcoma of bone

ELENI KAKOURI,[1] JEREMY S. WHELAN,[1] STEWART COLTART,[2] MARK E. F. SMITH[3] & ROBERT L. SOUHAMI[1]

[1]*The London Bone Tumour Service, The Middlesex Hospital, London,* [2]*Kent and Canterbury Hospital, Canterbury, Kent &* [3]*Department of Histopathology, University College Hospital, London, UK*

**Abstract**
A multi-focal multi-centric, malignant tumour of vascular origin arising in bone in a 55-year-old man is described. The presenting symptoms were pain and weight loss. Radiologically, multiple lytic lesions were demonstrated in the long bones of both legs and throughout the pelvis. Histological examination demonstrated an angiosarcoma which was predominantly low grade in nature but with focal areas of intermediate grade. Tumour cells expressed the endothelial markers CD31, CD34 and von Willebrand's factor. There was rapid radiological progression of disease with no response to radiotherapy. Pain abated within a few days of institution of doxorubicin, 75 mg $m^{-2}$, but the patient died of massive pulmonary thromboembolism 14 days later, 11 months after the first symptoms.

**Key words:** *angiosarcoma, CD31, CD34, von Willebrand's factor.*

### Introduction

Tumours of vascular origin arising in bone are rare, comprising fewer than 1% of primary bone tumours.[1] Difficulties exist in discriminating histologically benign vascular lesions from tumours which may pursue a more rapid, malignant course. Furthermore, the clinical course of such tumours may vary greatly. This has led to considerable confusion in terminology, particularly in the application of the term haemangioendothelioma to low-grade malignant vascular lesions and angiosarcoma to higher grade tumours. Mirra suggested that more than two-thirds of reported low-grade haemangioendotheliomas or angiosarcomas may in fact be benign haemangiomatous lesions.[2] The term, epithelioid haemangioendothelioma, introduced by Weiss and Enzinger for a group of soft tissue tumours of varying origin,[3] is now also sometimes used for primary tumours of bone.[4] Such tumours are said to pursue a relatively indolent course, metastasize rarely, and are distinguishable clinically and histologically from angiosarcoma.[4] We are encouraged to report the following patient because of the absence from the literature of a similar case and because it may illuminate further some of the areas of difficulty that surround this fascinating but uncommon group of tumours.

### Patient

In November 1993, a 55-year-old male smoker presented with a painful right calf. Over the next 8 months, his symptoms persisted and further pain developed in the right knee and left ankle. After an initial diagnosis of a muscle tear, he was treated with physiotherapy and immobilization in plaster. Radiographs of the right femur, tibia and fibula performed in December 1993 were thought to be normal. When the pain failed to improve, an arteriogram was performed which demonstrated occlusion of the right popliteal artery and he was referred for surgery. By July 1994, his pain had worsened, left ankle oedema developed and he had lost weight.

Investigations revealed a microcytic anaemia (Hb 11 g $dl^{-1}$), erythrocyte sedimentation rate (ESR) raised at 120 mm $h^{-1}$ impaired renal function (serum creatinine 386 $\mu$mol $l^{-1}$) and hypercalcaemia (corrected calcium 3.75 mmol $l^{-1}$). Parathyroid hormone levels, serum immunoglobulins and prostate specific antigen levels were normal. Bence Jones proteinuria was absent. A bone marrow trephine biopsy from the posterior iliac spine was normal.

A chest radiograph was also normal. Radiographs of the long bones of both legs showed multiple lytic lesions of varying size, more on the left than right (Figs 1 and 2). The tumour involved both femurs,

Correspondence to: J. Whelan, The London Soft Tissue & Bone Tumour Service, The Meyerstein Institute of Oncology, The Middlesex Hospital, Mortimer Street, London W1N 8AA, UK. Tel: + 44 (0)171 380 9092; Fax: + 44 (0)171 436 0160; E-mail: j.whelan@ucl.ac.uk.

1357-714X/97/030183-05 $9.00 © 1997 Carfax Publishing Ltd

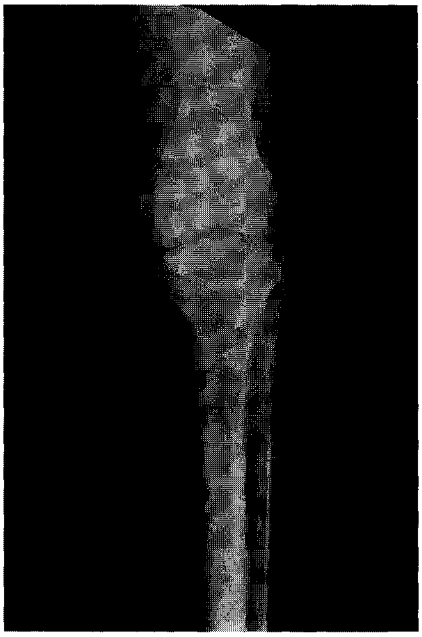

**Fig. 1.** *Radiograph of left leg showing multiple small lytic lesions of varying size distributed throughout the femur, tibia and fibula.*

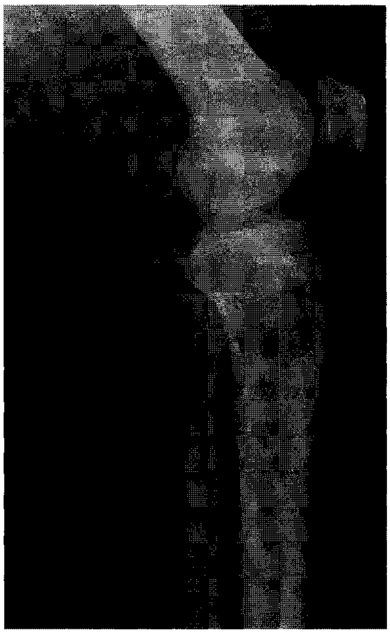

**Fig. 2.** *Overlapping lesions with indistinct borders indicating histologically aggressive disease.*

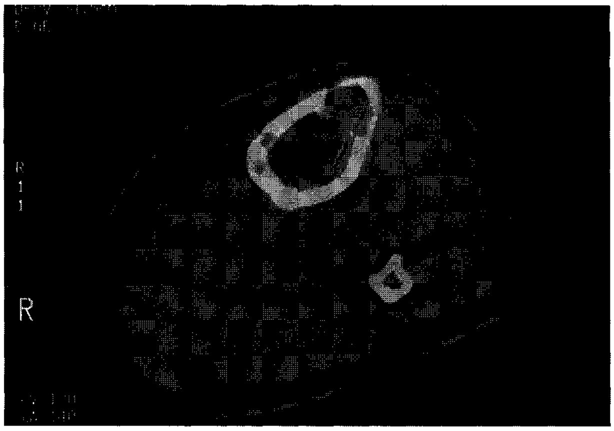

**Fig. 3.** *Computed tomogram through right tibia and fibula. The larger lesion is causing cortical destruction without expansion on a surrounding soft tissue mass.*

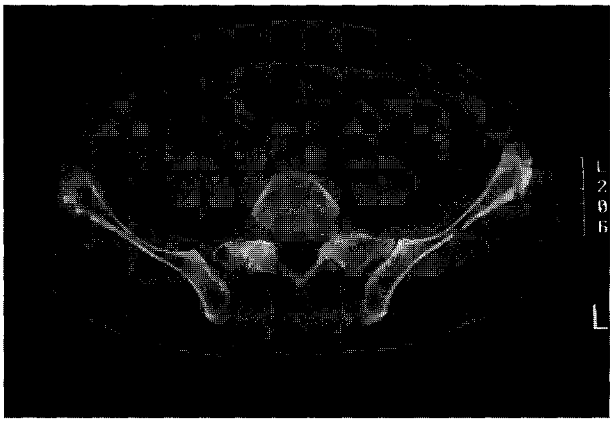

**Fig. 4.** *Multiple lesions were visible throughout the pelvis on computed tomography performed at presentation.*

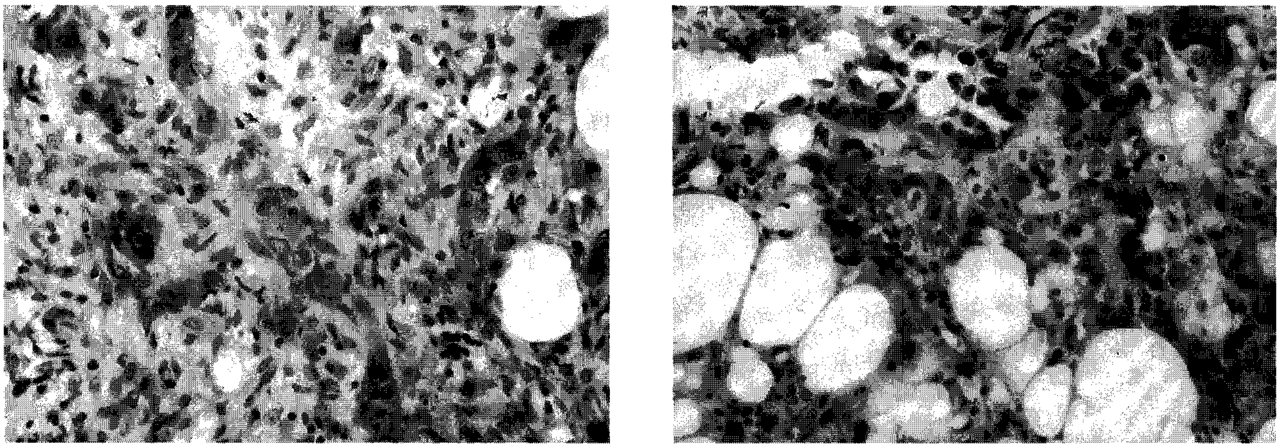

*(a)* *(b)*

**Fig. 5.** *Histology of the angiosarcoma showing well-developed neoplastic blood vessels lined by atypical endothelial cells. Intraluminal erythrocytes are visible in (a). Piling up of the endothelium and apparent shedding of cells into the lumen is seen in (b) (both haematoxylin & eosin,* × *400).*

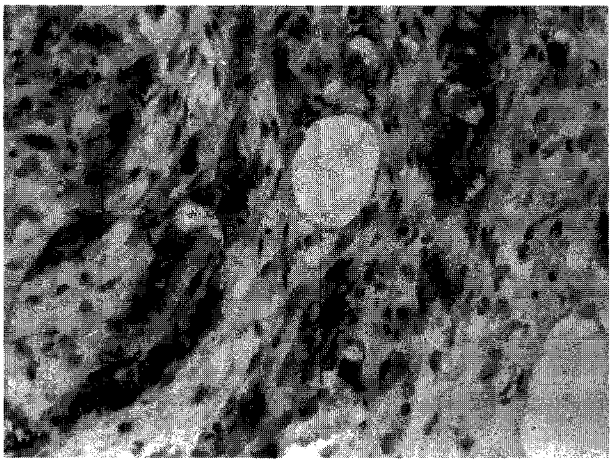

(c)

**Fig. 5.**

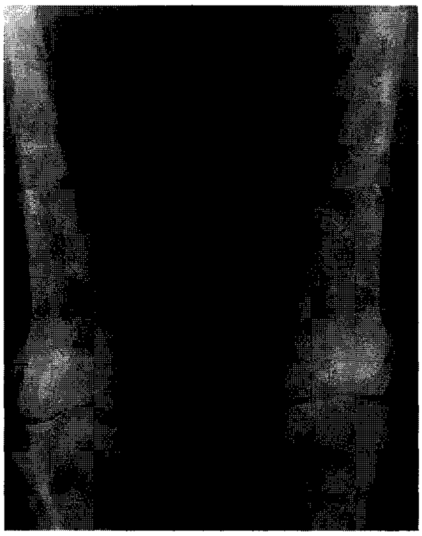

**Fig. 6.** *Extensive disease throughout the long bones of both legs, 2 months after diagnosis.*

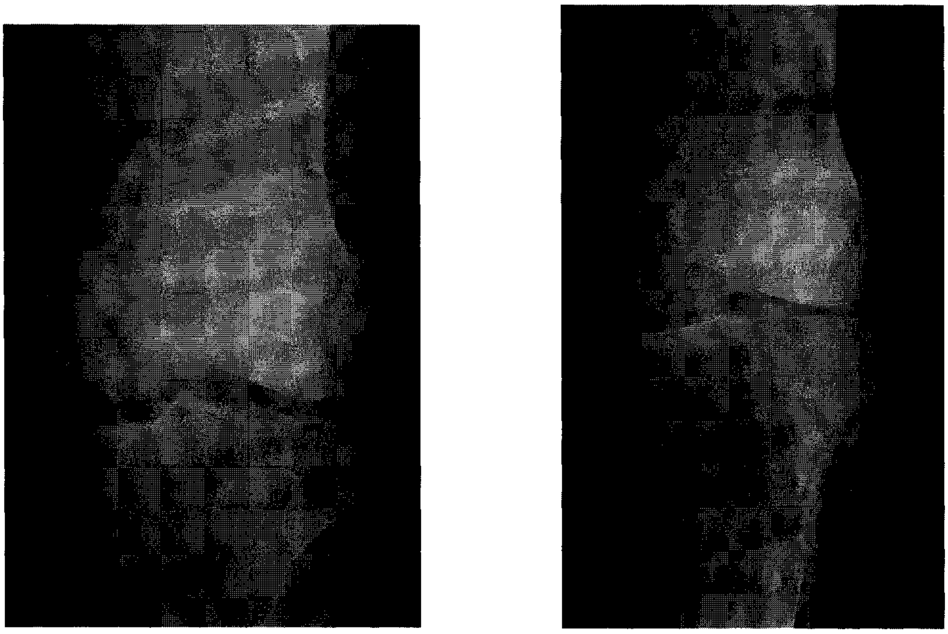

**Fig 7.** *Radiographs from July 1994 (left) and September 1994 (right) demonstrate rapid progression of disease.*

tibiae and fibulae. Some lesions had clearly defined margins, others very indistinct edges. There was no periosteal reaction but cortical destruction was seen. Some lesions appeared to overlap (Fig. 2). Review of X-rays taken in December 1993 revealed two 4-mm lytic lesions in the diaphysis of the right tibia. Similar lesions were also seen throughout the proximal femora and the pelvic bones, including the sacrum, on computed tomography (CT) (Figs 3 and 4). There were no adjacent soft tissue abnormalities. A 5-cm abdominal aortic aneurysm was noted but no visceral metastases were identified. Bone scan showed patchy uptake throughout the fibulae, tibiae and femora. Uptake was particularly intense around the left ankle and right knee.

Biopsy was performed of a representative lesion in the left tibia. Multiple tissue fragments were examined, revealing foci of angiosarcoma (Fig. 5). The angiosarcoma consisted of clusters of atypical cells with high nuclear-to-cytoplasmic ratios and, focally, pronounced nuclear pleomorphism. Tumour cells formed well-developed vascular channels within which many erythrocytes were identified (Fig. 5(a)). Tumour cells were often piled on top of each other and apparently shed into the lumen (Fig. 5(b)) Immunohistochemistry demonstrated expression of the endothelial markers CD31, CD34 and von Willebrand's antigen by neoplastic cells. The endothelial marker, cytokeratin, was not expressed. The tumour showed some foci of relatively low-grade histology with few mitoses and relatively little nuclear pleomorphism. However, in other areas, there were foci of substantial nuclear pleomorphism associated with considerable mitotic activity (4 per 10 high power fields) judged to be of intermediate histological grade.

Hypercalcaemia responded to pamidronate and renal function improved. In August 1994, prednisolone (10 mg day$^{-1}$) was started and radiotherapy (25 Gy) given to both tibiae but without symptomatic improvement. One week later the patient developed a confusional state, attributed to hyperglycaemia secondary to corticosteroids. Insulin was begun and his mental state improved. Radiological progression was evident (Figs 6 and 7). Doxorubicin (75 mg m$^{-2}$) was given and resolution of pain was noted within a few days but the patient died suddenly at home 14 days later, just 11 months from the first symptoms.

At autopsy, there was macroscopic evidence of thrombus in the calf veins of both legs, the cause of death being occlusion of both pulmonary arteries by thromboemboli. No evidence of visceral metastatic disease was seen. Tissue was not taken for histological examination nor was a more detailed examination made of calf and sacral venous plexi.

### Discussion

Several different terms have been applied to bone tumours of vascular endothelial origin since Stout first described haemangioendothelioma in 1943, and it is now possible to distinguish both clinical and pathological groupings within this diagnosis.[5] The benign course of many haemangioendotheliomas led to an adoption of the terms angiosarcoma or haemangiosarcoma to distinguish those more malignant tumours which display very disordered architecture of the characteristic complex, anastomosing vascular channels and a greater degree of pleomorphism.[6] Other investigators have suggested that tumours which follow an extremely benign course are in fact haemangiomas and that angiosarcoma should be the term adopted for all malignant tumours arising from vascular endothelium.[2,7] In our case, it was possible to demonstrate areas of varying histological grade even though only a single lesion was sampled. This feature may be generally under-recognized because of sampling constraints. However, variation in histological grade has not been reported consistently in histological examination of tumours treated with radical surgery when larger quantities of tissue are inspected.[6,8]

The most distinctive clinical feature of this disease is the occurrence of multiple bone lesions. These may be present in just one bone (multi-focal, solitary) or in adjacent bones (multi-focal, multi-centric, contiguous). The presence of lesions in several bones separated by more than one joint (multi-focal, multi-centric, non-contiguous) is exceptional and individual cases have never been very clearly documented.[2,6,9,10] Although, in this case, lesions were visible in all bones of both lower limbs, they were also seen in the pelvis and sacrum, making this a rather unusual example of contiguous disease.

The distribution of lesions raises questions as to how this disease spreads. It has been postulated that spread to contiguous bones may be via metaphyseal veins, with tumour detectable in both large and small veins, a process that may occur relatively slowly.[2] Termed angiotropism, both antegrade and retrograde spread can occur. In this case, it is possible that retrograde spread, via the pelvic venous plexus, is the mechanism by which lesions appeared in both tibiae and fibulae although no pathological confirmation of venous involvement was made. We believe that although the distribution of disease described in this report fits with Mirra's definition of contiguity, more supporting evidence for such a remarkable process of dissemination is needed to account safely for such widespread involvement and rapid progression. The predilection of this tumour for bone remains unexplained.

Extraosseous metastases are occasionally seen late in the course of patients presenting with solitary angiosarcomas of bone but have not been described in multi-focal disease. Two points are worthy of emphasis with regard to this case: first, there was no evidence of pulmonary metastases at autopsy; second, there was evidence of thrombosis in the calf venous plexus of both legs with death being caused by pulmonary embolism. It is tempting to speculate whether these were in fact tumour emboli, as seen in other primary bone tumours and choriocarcinoma.[11–13]

Numerous authors have commented that multifocal disease pursues a more indolent course and carries a more favourable prognosis than that associated with a single lesion, when metastases, most often to the lungs, may occur with great rapidity.[10,14–16] This has been disputed by some who instead have stressed the prognostic importance of histological grade as a more useful determinant of outcome than the number of lesions.[8,9] Although no high-grade tumour was seen in the biopsy material, certain clinical features indicate that the tumour was more aggressive. The progression of disease radiologically was rapid over the 11-month period of observation. Individual lesions varied in size, clarity of margin and associated cortical destruction. No sclerosis or 'soap bubble' appearance of ballooning periosteum was evident, both said to predict for a more indolent course, but neither was there periosteal reaction or evidence of soft tissue disease, which are radiological features of higher grade disease.[8,9,17,18] Finally, the patient developed marked hypercalcaemia, previously reported only once in a patient with the multi-focal, contiguous variant.[15]

Management of malignant vascular tumours is primarily surgical but recurrence after curettage alone is common. Amputation may still be curative in patients with recurrent tumours, even if multicentric.[2,15,19] Campanacci *et al.* has recommended that grade I lesions are treated by curettage alone, grade II lesions by resection sometimes with radiotherapy, and that Grade III tumours are treated aggressively with ablative surgery and chemotherapy. Caution must be applied to the application of such guidelines as differences in the clinical course clearly existed within the groups.[8] Radiotherapy has been widely applied and appears to be effective for local control in some patients,[9,19,20] occasionally with dramatic benefit.[21,22] There are no reports of the efficacy of chemotherapy in the literature although some authors have recommended its use for angiosarcoma.[8,9] In this case, there was no symptomatic response to radiotherapy but there was rapid resolution of pain soon after chemotherapy began.

### Acknowledgements

The authors are grateful for the comments of Dr C. Lawson and of Dr J. M. Mirra.

### References

1 Dorfmann HD, Czerniak B. Bone cancers. *Cancer* 1995; 75:203–10.
2 Mirra JM. Angiosarcoma of bone: solitary and multifocal variants. In: Mirra JM, Picci P, Gold RH, eds. *Bone tumours, Clinical, radiologic and pathologic correlations*. Philadelphia: Lea and Febiger, 1989:1382–417.
3 Weiss SM, Enzinger FM. Epithelioid hemangioendothelioma: a vascular tumor often mistaken for a carcinoma. *Cancer* 1982; 50:970–81.
4 Fechner RE, Mills SE. Tumours of the bones and joints. In: *Atlas of tumor pathology*. Washington, DC: Armed Forces Institute of Pathology, 1993:135–40.
5 Stout AP. Hemangio-endothelioma: a tumour of blood vessels featuring vascular endothelial cells. *Ann Surg* 1943; 118:445–64.
6 Volpe R, Mazabraud A. Hemangioendothelioma (angiosarcoma) of bone: a distinct pathologic entity with an unpredictable course? *Cancer* 1982; 49:727–36.
7 Rosai J, Gold J, Landy R. The histiocytoid hemangiomas: a unifying concept embracing several previously described entities of skin, soft tissue, large vessels, bone and heart. *Hum Pathol* 1979; 10:707–30.
8 Campanacci M, Boriani S, Giunti A. Hemangioendothelioma of bone: a study of 29 cases. *Cancer* 1980; 46:804–14.
9 Wold LE, Unni KK, Beabout JW, *et al.* Hemangioendothelial sarcoma of bone. *Am J Surg Pathol* 1982; 6:59–70.
10 Tsuneyoshi M, Dorfman HD, Bauer TW. Epithelioid hemangioendothelioma of bone: a clinical, ultrastructural and immunohistochemical study. *Am J Surg Pathol* 1986; 10:754–64.
11 Warren S. Chondrosarcoma with intravascular growth and tumour emboli to lungs. *Am J Pathol* 1931; 7:161–7.
12 Bagshawe KD, Brooks WD. Subacute pulmonary hypertension due to horioepithelioma. *Lancet* 1959; i:653–8.
13 Wakasa K, Sakurai M, Uchida A, *et al.* Massive pulmonary tumor emboli in osteosarcoma. *Cancer* 1990; 66:583–6.
14 Hartmann WH, Stewart FW. Hemangioendothelioma of bone. *Cancer* 1962; 15:846–54.
15 Otis J, Hutter RV, Foote FW, *et al.* Hemangioendothelioma of bone. *Surg Gynecol Obstet* 1968; 127:295–305.
16 Huvos AG. *Bone tumours: diagnosis, treatment and prognosis*. Philadelphia: WB Saunders, 1979:358–68.
17 Unni KK, Ivins JC, Beabout JW, *et al.* Hemangioma, hemangioperiytoma, and hemangioendothelioma (angiosarcoma) of bone. *Cancer* 1971; 27:1403–14.
18 Abrahams TG, Bula W, Jones M. Epithelioid hemangioendothelioma of bone. *Skeletal Radiol* 1992; 21:509–13.
19 Larsson S, Lorentzon R, Boquist L. Malignant hemangioendothelioma of bone. *J Bone Joint Surg* 1975; 57A:84–9.
20 Senan S, Canney PA. Failure of palliative radiotherapy in high-grade angiosarcoma of bone. *Eur J Cancer* 1992; 28A:1934.
21 Morgenstern P, Westing SW. Malignant hemangioendothelioma of bone. Fourteen year follow up in a case treated with radiation alone. *Cancer* 1969; 23:221–4.
22 Chow RL, Wilson CB, Olsen ER. Angiosarcoma of the skull. Report of a case and review of the literature. *Cancer* 1970; 25:902–6.

## References

[PDF_00327] Abrahams T. G., Bula W., Jones M. (1992). Epithelioid hemangioendothelioma of bone. A report of two cases and review of the literature.. Skeletal Radiol.

[PDF_00311] BAGSHAWE K. D., BROOKS W. D. (1959). Subacute pulmonary hypertension due to chorionepithelioma.. Lancet.

[PDF_00298] Campanacci M., Boriani S., Giunti A. (1980). Hemangioendothelioma of bone: a study of 29 cases.. Cancer.

[PDF_00339] Chow R. W., Wilson C. B., Olsen E. R. (1970). Angiosarcoma of the skull. Report of a case and review of the literature.. Cancer.

[PDF_00274] Dorfman H. D., Czerniak B. (1995). Bone cancers.. Cancer.

[PDF_00317] HARTMANN W. H., STEWART F. W. (1962). Hemangioendothelioma of bone. Unusual tumor characterized by indolent course.. Cancer.

[PDF_00330] Larsson S. E., Lorentzon R., Boquist L. (1975). Malignant hemangioendothelioma of bone.. J Bone Joint Surg Am.

[PDF_00336] Morgenstern P., Westing S. W. (1969). Malignant hemangioendothelioma of bone. Fourteen-year follow-up in a case treated with radiation alone.. Cancer.

[PDF_00319] Otis J., Hutter R. V., Foote F. W., Marcove R. C., Stewart F. W. (1968). Hemangioendothelioma of bone.. Surg Gynecol Obstet.

[PDF_00294] Rosai J., Gold J., Landy R. (1979). The histiocytoid hemangiomas. A unifying concept embracing several previously described entities of skin, soft tissue, large vessels, bone, and heart.. Hum Pathol.

[PDF_00333] Senan S., Canney P. A. (1992). Failure of palliative radiotherapy in high-grade angiosarcoma of bone.. Eur J Cancer.

[PDF_00288] Stout A. P. (1943). HEMANGIO-ENDOTHELIOMA: A TUMOR OF BLOOD VESSELS FEATURING VASCULAR ENDOTHELIAL CELLS.. Ann Surg.

[PDF_00304] Tsuneyoshi M., Dorfman H. D., Bauer T. W. (1986). Epithelioid hemangioendothelioma of bone. A clinicopathologic, ultrastructural, and immunohistochemical study.. Am J Surg Pathol.

[PDF_00324] Unni K. K., Ivins J. C., Beabout J. W., Dahlin D. C. (1971). Hemangioma, hemangiopericytoma, and hemangioendothelioma (angiosarcoma) of bone.. Cancer.

[PDF_00291] Volpe R., Mazabraud A. (1982). Hemangioendothelioma (angiosarcoma) of bone: a distinct pathologic entity with an unpredictable course?. Cancer.

[PDF_00314] Wakasa K., Sakurai M., Uchida A., Yoshikawa H., Maeda A. (1990). Massive pulmonary tumor emboli in osteosarcoma. Occult and fatal complication.. Cancer.

[PDF_00308] Warren S. (1931). Chondrosarcoma with Intravascular Growth and Tumor Emboli to Lungs.. Am J Pathol.

[PDF_00282] Weiss S. W., Enzinger F. M. (1982). Epithelioid hemangioendothelioma: a vascular tumor often mistaken for a carcinoma.. Cancer.

[PDF_00301] Wold L. E., Unni K. K., Beabout J. W., Ivins J. C., Bruckman J. E., Dahlin D. C. (1982). Hemangioendothelial sarcoma of bone.. Am J Surg Pathol.

